# A212 THE IMPACT OF COMBINED MEDICAL AND SURGICAL TREATMENT FOR PERIANAL CROHN’S DISEASE: A SYSTEMATIC REVIEW AND META-ANALYSIS

**DOI:** 10.1093/jcag/gwac036.212

**Published:** 2023-03-07

**Authors:** M Fung, Y Farbod, H Kankouni, J McCurdy

**Affiliations:** 1 Division of Gastroenterology and Hepatology, Department of Medicine; 2 Department of Medicine, University of Ottawa; 3 Ottawa Hospital Research Institute, Ottawa, Canada

## Abstract

**Background:**

Multidisciplinary care involving surgical drainage via examinations under anesthesia (EUA) and anti-tumor necrosis factor (TNF) therapy is recommended for perianal Crohn’s disease (PCD). However, the impact of this combined modality approach is not well-established.

**Purpose:**

To determine the impact of combined modality (surgical and anti-TNF) vs. single modality (either surgical or anti-TNF) therapy on fistula healing in perianal Crohn’s disease.

**Method:**

MEDLINE, EMBASE, and Cochrane databases were searched systematically from inception through September 15, 2022 independently in duplicate. We included studies that reported on fistula outcomes after treatment with combined modality vs. single modality therapy for PCD. Surgery was defined as EUA ± seton or closure procedure. Weighted summary estimates with 95% CI were estimated by random effects models. Study quality was determined using an adapted version of the Newcastle-Ottawa scale.

**Result(s):**

Thirteen studies met inclusion criteria. The total population included 1279 patients: 685 patients treated with single modality therapy with either surgical or anti-TNF therapy and 594 patients treated with combined modality therapy. Patients treated with combined modality therapy were more likely to achieve fistula healing compared to single modality therapy (65.4% vs. 57.7%; OR 1.68; 95% CI 1.03-2.73, p = 0.04) (Figure 1a). In a subgroup analysis, the rates of fistula healing were higher with combined modality therapy than with surgical therapy alone (OR 2.34; 95% CI 0.85-6.46, p = 0.10) but not anti-TNF therapy alone (OR 1.19; 95% CI 0.78-1.81, p = 0.43), although neither comparison was statistically significant (Figures 1b and c). The majority of studies were rated low-quality due to risk of bias from uncontrolled confounding variables.

**Image:**

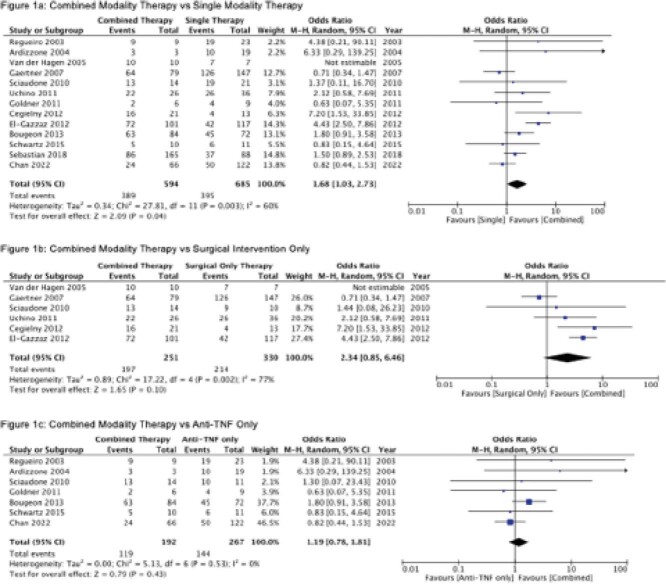

**Conclusion(s):**

Limited high-quality evidence suggests that fistula healing occurs more frequently in patients treated with combined modality therapy. However, the benefit of a combined modality approach appears to be driven mainly by anti-TNF therapy. Further prospective randomized trials are needed to confirm these findings.

**Please acknowledge all funding agencies by checking the applicable boxes below:**

None

**Disclosure of Interest:**

None Declared

